# Newly diagnosed cirrhosis secondary to gastrointestinal bleed due to portal hypertensive colopathy

**DOI:** 10.1093/jscr/rjad114

**Published:** 2023-03-07

**Authors:** Nestor Sabat, Bettina Schulze

**Affiliations:** Department of General Surgery, Mackay Base Hospital, Mackay, Australia; Department of General Surgery, Mackay Base Hospital, Mackay, Australia

**Keywords:** portal hypertension, portal hypertensive colopathy, cirrhosis complication

## Abstract

Portal hypertensive colopathy (PHC) is a colonic phenomenon commonly causing chronic gastrointestinal bleeding or less commonly a life-threatening acute colonic hemorrhage. An otherwise well, 58-year-old female presents general surgeons a diagnostic dilemma for symptomatic anemia. An interesting case where the rare and elusive PHC was diagnosed on colonoscopy, which led to the diagnosis of liver cirrhosis without evidence of oesophageal varices. Although PHC is most common in patients with cirrhosis, it is likely still underdiagnosed, given the current stepwise treatment approach of these cirrhotic patients often leads to treatment of the PHC alongside PHG without establishing a diagnosis. Instead, this case presents a generalised approach to patients with underlying portal and sinusoidal hypertension due to a variety of causes, and the endoscopic and radiological findings, which lead to their successful diagnosis and medical management of the gastrointestinal bleeding.

## INTRODUCTION

Portal hypertensive colopathy (PHC) is a colonic phenomenon commonly causing chronic gastrointestinal bleeding or less commonly a life-threatening acute colonic hemorrhage. Described since 1991, the literature suggests portal and sinusoidal hypertension are essential for developing characteristic macroscopic changes including vascular ectasias, anorectal or colonic varices, hemorrhoids and nonspecific inflammatory [1]. Microscopically dilatation of capillaries and venues in mucosa and submucosa with or without inflammation are important features but often difficult to observe in histopathology [1, 2]. The underlying causes for these changes reported to date in literature include portal vein thrombosis, schistosomiasis, veno-occlusive disease, cardiac failure and, most notably, liver cirrhosis [1, 3]. Liver cirrhosis patients with portal hypertension have the highest prevalence of PHC (20–98%) [1, 4]. Additionally, PHC is often diagnosed following the more well-known and prevalent portal hypertensive gastropathy (PHG), which leads to esophageal varices reflecting advanced cirrhosis. [1, 4] Medical treatment for both of these conditions includes iron replacement, blood transfusions and portal pressure-reducing agents like octreotide [1, 5].

## CASE REPORT

A 58-year-old female presents an interesting diagnostic dilemma of symptomatic anemia, describing 6 weeks of painless intermittent bleeding per rectum alongside dizziness and crippling fatigue on the background of a colonoscopy performed 3 years prior for a positive FOBT showing diverticulosis, colonic polyps, grade-1 hemorrhoids and a friable caecum. The histopathology showed non-specific focal active colitis and benign polyps. In this presentation, she denies any hematemesis, melena, changes in bowel habits, nausea or vomiting, anorexia or any B symptoms. Her past medical and surgical history were unremarkable denying tobacco use or substance misuse intravenously but reported consuming eight standard drinks per day, for years. The abdominal examination was quite unremarkable with stable vital signs, no stigmata of liver disease and an unremarkable digital rectal exam without evidence of bleeding, hemorrhoids or perianal disease.

**Figure 1:**
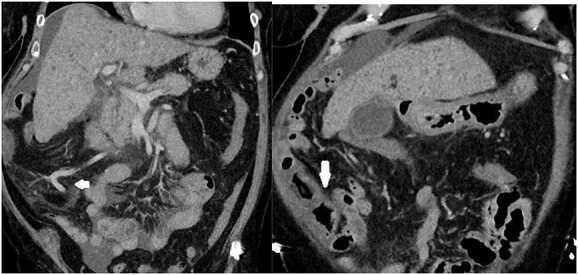
Images depicting a single slice of the CT Abdomen/Pelvis with contrast completed on admission. Arrows pointing to subtly dilated mesenteric vessels, as well as thickened bowel affected by PHC, is depicted in Fig. 2.

**Figure 2:**
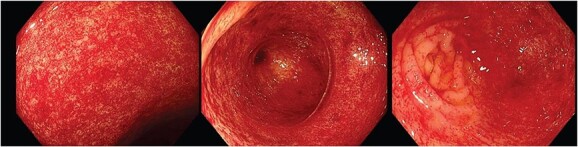
Endoscopic images depicting macroscopic findings of PHC including vascular ectasias and nonspecific inflammatory mucosal changes leading to friable mucosa which bled on contact.

Given initial biochemical markers suggested a significant bleeding diathesis (Hemoglobin 55 g/L(Reference (120–160 g/L).); low platelets (88 × 10^9^/ L)(Reference (150–450 x 10^9^/L).); elevated INR (1.5)(Reference (<1.0).); and normal liver function tests, urea and inflammatory markers), she was taken for a computed tomography (CT)-angiogram of the abdomen and pelvis. A focus of active bleeding on all phases was not found with the radiologist reporting non-specific porta hepatis oedema without any evidence of cirrhosis, intrabdominal ascites, or vascular thrombus (Fig. 1). Despite adequate medical optimisation and blood transfusions the next day she collapsed and deteriorated acutely due to a presumed acute bleed, given her CT head was unremarkable. After intense resuscitation, the patient was taken to theater emergently where the gastroscopic findings were unremarkable and varices were not identified, whereas the colonoscopy found significant severe inflammation, a large adherent blood clot, congestion (oedema) and caecal erythema (Fig. 2). At this time, the radiologist’s report for the liver ultrasound performed immediately before the endoscopic procedures confirmed a new diagnosis of liver cirrhosis. After adequate further medical investigation and optimisation of her liver cirrhosis, an iron transfusion and 3 days of a continuous octreotide infusion, she went home for further outpatient management of her liver disease without any further stigmata of bleeding to date.

## DISCUSSION

This is a rare case of PHC diagnosed on colonoscopy for acute intestinal bleeding, leading to a new diagnosis of liver cirrhosis reflecting an important diagnostic and management dilemma. Unlike the repeated endoscopic assessment and treatment of esophageal varices, surveillance colonoscopies assessing PHC are not performed and explain the variability in prevalence as many remain underdiagnosed [1]. Additionally, if the diagnosis of liver cirrhosis was known in this case, then esophageal varices would have been the most likely diagnosis, leading to preemptive medical and endoscopic management, which would treat both the PHG and PHC, leaving PHC undiagnosed as the intestinal bleeding would be resolved. Interestingly, this case presents a cascade of events that are in reverse to those previously presented in literature. Liver cirrhosis was undiagnosed, no esophageal varices were found and initial, therefore, medical management did not include octreotide but instead lead to the initial diagnosis of PHC, which led to medical treatment of presumed liver cirrhosis that was subsequently confirmed on a liver ultrasound.

Figure 2 depicts the macroscopic findings encountered that led to this exceptional diagnosis of PHC by an experienced endoscopist. The endoscopic mucosal changes are non-specific inflammation and subtle vascular changes, which are important features described in literature [1]. These were noted by the histopathologist microscopically but were unable to provide a diagnosis. This scenario often presents many endoscopists and histopathologists a significant challenge as the variations in endoscopic descriptions and the absence of uniform diagnostic criteria/classifications in literature can make a definitive diagnosis especially difficult. In these cases, clinicians often rely on patient history to identify a possible underlying cause for the endoscopic findings, such as alcohol misuse.

Instead, this case presents a strong argument for the use of CT from the armamentarium of general surgeons in the diagnosis of PHC because it provided invaluable clues depicted in Fig. 1. Although initially not identified, the radiologists subsequently commented that the mesenteric vessels showed significant venous dilatation without fat stranding to suggest widespread inflammation or infection. Instead, the bowel draining into these vessels displayed a thicker wall suggesting dilatation and possibly venous congestion contributing to the bowel thickening and non-specific inflammation. This evidence of portal hypertension coupled with endoscopic findings led to successful diagnosis and management of PHC before the diagnosis of cirrhosis on ultrasound.

## CONCLUSION

An interesting case where the rare and elusive PHC was diagnosed on colonoscopy led to the diagnosis of liver cirrhosis without evidence of esophageal varices. Although PHC is most common in patients with cirrhosis, it is likely still underdiagnosed, given the current stepwise treatment approach of these cirrhotic patients often leads to the treatment of the PHC alongside PHG without establishing a diagnosis. Instead, this case presents a generalised approach to patients with underlying portal and sinusoidal hypertension due to a variety of causes, and the endoscopic and radiological findings that lead to their successful diagnosis and medical management of the gastrointestinal bleeding.

## DISCLOSURE STATEMENT

There are no sources of funding, financial support or industrial affiliations to disclose. The authors declare that they have no conflicts of interest relevant to the manuscript submitted to *Journal of Surgical Case Reports*.

All authors are in agreement with the contents of the manuscript and confirm that the paper is not being published or under consideration elsewhere.

## Data Availability

Data sharing is not applicable to this article as no new data was created or analyzed in this study.
